# Tramadol Versus Codeine in Hand Surgery

**DOI:** 10.7759/cureus.26886

**Published:** 2022-07-15

**Authors:** Jacob Tulipan, Jack Abboudi, Mark L Wang, Moody Kwok, Daniel Seigerman, Greg G Gallant, Pedro Beredjiklian

**Affiliations:** 1 Division of Hand Surgery, Rothman Orthopaedic Institute, Philadelphia, USA; 2 Orthopaedic Surgery, Rothman Orthopaedic Institute, New York, USA

**Keywords:** pain management, opioid, hand surgery, codeine, tramadol

## Abstract

Introduction

Tramadol and codeine are both commonly prescribed in the setting of surgery or injury to the upper extremity. Despite their comparable strength in terms of opioid receptor affinity, the drugs differ pharmacologically and thus are not completely interchangeable.

Methods

This study analyzes all prescriptions for codeine and tramadol by a group of hand surgeons over a one-year period and tests the central hypothesis that the prescribing and refill patterns of these two drugs would be similar.

Results

Despite similar prescription amounts in terms of morphine equivalents, patients receiving tramadol required prescription refills at a significantly higher amount than those receiving codeine, and these individuals tended to be older. Additionally, patients treated nonoperatively were prescribed significantly more tramadol than those treated surgically.

Conclusion

Our findings suggest that codeine and tramadol are not equivalent in managing upper extremity pain. Further study is needed to articulate the situations in which physicians and patients are better served by tramadol versus codeine.

## Introduction

Despite the ongoing opioid crisis in the United States, opioids continue to remain a mainstay of perioperative and nonoperative pain management [1]. While not an opioid, tramadol is converted in the body to an active metabolite with Mu opioid receptor affinity, and thus, has opioid-like adverse effects, notably respiratory depression and dependence [2]. Tramadol, despite its status as a “semi-opiate,” has a true dependency and abuse potential and undergoes a complex series of drug-drug interactions [3]. As an opioid with generally low strength as measured in morphine milligram equivalents (MMEs), tramadol (0.1 MME) occupies much the same niche as codeine (0.15 MME) in treating pain that does not require higher potency opioids [4]. Indeed, a 2006 study on chronic pain patients found a rate of abuse (0.7%) more closely matching that of non-steroidal anti-inflammatory drugs (NSAIDs) (0.5%) and hydrocodone (1.2%) [5]. The frequency of prescribing tramadol has increased markedly since 2005 [6], likely at least in part because it is perceived to have fewer risks than a true opioid.

Considering the ongoing effort to understand the prescription patterns for opioids, the relative use patterns of tramadol and codeine, which are seemingly comparable pain-controlling medications, warrant further investigation. The purpose of this study is to evaluate the tramadol and codeine prescription patterns and refill patterns for patients whose pain is managed by hand surgeons, to test the hypotheses that prescribing patterns for tramadol and codeine would be equivalent, would yield equivalent rates of refills, and would demonstrate equivalent MME prescription amounts.

## Materials and methods

This study is an IRB-approved review of patients from 18 fellowship-trained hand surgeons at a regional private/academic practice. Inclusion criteria consisted of all patients evaluated and treated with encounter dates during the calendar year 2021 who were prescribed at least one controlled substance. No patients were excluded. A chart review identified patients prescribed medications containing codeine or tramadol as ordered through the electronic medical record. Patients were grouped as being prescribed either tramadol or codeine, as these medications are similar in their relative morphine equivalency and are used in our practice for the management of pain following similar procedures. No patients received prescriptions for both medications. Each surgeon prescribed the medication per their usual treatment routine and discretion. Codeine prescriptions were combined with acetaminophen as is commercially available and contained 30 mg of codeine and 300 mg of acetaminophen. Tramadol prescriptions included those from tramadol alone as well as those for a combination of tramadol (37.5 mg) and acetaminophen (325 mg). Patients without contraindications were instructed to supplement these medications with NSAIDs.

The total amount of MME prescribed for each patient, including the initial prescription and up to two refills, was calculated in milligrams using the MME conversions as outlined by the US Department of Health and Human Services [4], with tramadol calculated as 0.1 and codeine calculated as 0.15 times the relative strength of morphine. Demographic data on each patient, including age and sex, were obtained via chart review. A chart review was also performed to determine whether each patient was treated surgically or nonoperatively. Tramadol versus codeine as well as operative versus nonoperative groups were compared with a two-tailed student’s t-test for continuous variables, while the chi-square test was used for categorical variables. Differences were considered significant at a p<0.05.

## Results

A total of 2433 prescriptions in 2025 patients were identified who met the inclusion criteria. Demographics are shown in Table 1.

**Table 1:** Demographic information for patients in tramadol and codeine groups. Age is listed as mean (range).

	Tramadol	Codeine
Total patients	1044	1389
Age	58.8 (16-96)	54.1 (10-96)
Sex	56% Female	53% Female
Surgically treated	929 (89%)	1239 (89%)

Of these prescriptions, 1389 (57.1%) were for codeine and 1044 (42.9%) were for tramadol. All codeine prescriptions were sent by board-certified hand surgeons or their physician assistants and were for codeine/acetaminophen 30/300 mg tablets. About 26 tramadol prescriptions (2.5%) were for tramadol/acetaminophen 37.5/325 mg tablets, the remaining 1018 (97.5%) were for tramadol alone. A total of 200 refills for codeine (14.4% refill rate), and a total of 181 refills for tramadol (17.3% refill rate) were sent. The overall rate of refills was statistically higher for tramadol (p=0.045).

Patients who were prescribed tramadol received significantly more MME than patients receiving codeine (70 mg versus 63.5 mg, p=0.003) when considering both those who did and did not obtain refills. In patients who did not require prescription refills, equivalent total MMEs per prescription were sent (62.9 mg for codeine versus 60.8 mg for tramadol, p=0.3). However, patients who were prescribed tramadol and went on to require a refill were prescribed more total opioids than patients who were prescribed codeine and went on to require a refill (114.4 mg MME versus 66.5 mg MME, p<0.0001).

In the codeine group, patients who required a refill did not differ significantly in initial prescription size from those who did not require a refill (62.9 versus 66.5). In the tramadol group, patients requiring a refill had been given significantly larger initial prescriptions for tramadol (114.4 mg versus 60.8 mg, p=0.0001).

In each group, 89% of prescriptions were given to patients who had undergone a surgical procedure. Surgical patients received similar total MME regardless of group (62.9 for codeine versus 67.4 for tramadol, p=0.2). In the codeine group, surgical and nonsurgical groups did not differ significantly in the average total MME per prescription (62.9 versus 67.7, respectively). The surgical patients receiving tramadol received significantly fewer MME than those treated nonoperatively (67.4 versus 90.9, p=0.0002). About 178 of 929 (19.2%) surgical patients who were prescribed tramadol required a medication refill, while 193 of 1239 (15.6%) surgical patients receiving codeine required a medication refill. This difference was statistically significant (p=0.028).

## Discussion

This study’s results did not support our hypothesis that prescriptions for codeine and tramadol in hand pathology would not differ significantly with regard to the total prescribed morphine equivalent. Our study demonstrated a greater average total MME prescribed for patients receiving tramadol than in those prescribed codeine. Additionally, there was a slightly higher refill rate in patients receiving tramadol than in those receiving codeine. The codeine and tramadol groups were similar in the total number of MMEs prescribed to operative patients, however, nonoperative patients receiving tramadol were given prescriptions for 23.2 mg MME greater total doses on average. In addition, the group prescribed tramadol was an average of 4.7 years older than the group prescribed codeine.

The greater average total prescribed MME in patients receiving tramadol is only noted when considering patients who received refills rather than initial prescriptions only. It can be inferred that the greater total prescribed MME of tramadol may have been driven by the increased rate of refill prescriptions in this group. Possible explanations for the increased rate of refills prescribed for the tramadol group could include the possibility that tramadol is not as effective as codeine in controlling these patients’ pain, and that the side effects of codeine make it comparatively less palatable for the patient to request a refill, or that the drugs are being prescribed to different patient populations who have different rates of requests for refills. The higher total MME of tramadol prescribed to nonoperative patients supports the latter hypothesis. To assist in establishing the reasons for higher refill rates in tramadol prescriptions, further studies would benefit from correlating visual analog scale (VAS) pain score with pathology and prescribed analgesic.

Pain after hand surgery or injury varies significantly based on diagnosis, etiology, and patient factors. In a number of the most common procedures, tramadol and codeine are frequently used for acute postoperative pain relief [7]. A prior study compared tramadol and opioids (including codeine) in postoperative pain relief after the carpal tunnel and found tramadol and opioids to be equally effective [8]. Despite these apparent similarities, tramadol does differ from opioids in some respects, including its reliance on cytochrome P450 2D6 to be converted to the active form O-desmethyltramadol (M1) [9], as well as its function as a serotonin and norepinephrine reuptake inhibitor [2]. 

The equivalent total prescription MME of tramadol and codeine given to operative patients is consistent with their rough equivalence in potency, while the disparity in total prescription MME given to nonoperative patients is not. This may be due to the perception of tramadol as a non-opioid or semi-opioid, resulting in tramadol being prescribed more liberally for subacute or chronic conditions or in patients who report opioid allergy or intolerance, although tramadol itself may cause nausea, drowsiness, and constipation frequently seen in patients with opioid intolerance [1]. The perception of tramadol as a non-opioid or semi-opioid may also factor into the preferential choice for prescribing this medication when there may be an expectation of a longer duration of treatment. This is supported by the fact that patients who had high initial prescription amounts for tramadol (i.e., those with the expectation of longer periods of requiring tramadol) were more likely to receive refills.

Patients prescribed tramadol after a surgical procedure were noted to be significantly more likely to require a refill of narcotics, despite the equivalent initial MME prescription being given. This may indicate that tramadol is less effective at relieving pain in these patients, that physicians are more likely to refill tramadol prescriptions, that the side effect profile of tramadol is better tolerated, and thus the patient may be more apt to request a refill. Note that tramadol’s dependency on CYP2D6 for effect has real-work implications in its efficacy. A study by Frost et al. demonstrated that inpatients receiving strong CYP2D6 inhibitors (including common drugs such as fluoxetine, bupropion, and quinidine) were more likely to experience breakthrough pain when prescribed tramadol [10]. Further study would be required to determine whether our findings of increased refill needs in tramadol are affected by patients' concurrent use of CYP2D6 inhibitors.

Alternatively, this finding may be due to practice considerations on the part of the prescriber. In a patient who appears to have a higher risk of dependence or abuse, or in whom analgesia requirements are higher, there is some evidence that tramadol has a lower abuse potential than opioids [5].

The use of tramadol in chronic musculoskeletal pain is controversial. A 2019 Cochrane systematic review of 22 randomized controlled trials by Toupin April et al. found moderate-quality evidence that tramadol had no important benefit on pain reduction in osteoarthritis versus placebo alone, and in fact, the side effects of tramadol lead to a higher rate of treatment discontinuation in tramadol versus placebo groups [11]. Long-term use of tramadol may result in paradoxical hyperalgesia [12] withdrawal, and abuse. Although initial published data indicated a low abuse potential for tramadol [13], a survey of nearly 70,000 people demonstrated a lifetime risk of oral tramadol misuse increasing from 0.4% in 2002 to 1.5% in 2014 [14]. The acute withdrawal syndrome for tramadol combines the sweating and gastrointestinal hyperactivity of opioid withdrawal with the anxiety, confusion, and psychiatric manifestations of selective serotonin reuptake inhibitor (SSRI) withdrawal such as delusions and hallucinations, as would be expected given the drug’s combined mechanism of action [15]. Further risks of long-term tramadol use are still under investigation: a 2021 study by Xie et al. retrospectively reviewed charts of over one million patients with prescriptions for tramadol or codeine and found that patients given tramadol had a higher risk of subsequent mortality, cardiovascular events, and fractures [16].

The perception of tramadol as a safer alternative to codeine may also be responsible for the greater average age of patients prescribed tramadol. There is some evidence to support this perception: a 2010 study on nonmalignant pain demonstrated an elevated risk of cardiovascular events and fracture in elderly patients prescribed codeine versus tramadol [17]. However, a subsequent study demonstrated a higher risk of hip fracture in patients given tramadol versus codeine [18].

This study is limited by its retrospective and nonrandomized nature. As a retrospective chart review, we were unable to assess how many pills were taken by each patient, only how many prescriptions were prescribed and refilled. There is evidence that many postoperative analgesics prescribed after hand surgery are not used [8]. Although patients were generally instructed to use acetaminophen if given a tramadol formulation not containing this drug, patients who nonetheless took tramadol alone may have had less effective analgesia. Further, because the prescription of tramadol versus codeine was left up to the treating physician, there is a clear source of selection bias that would be expected to affect the demographics and pain medication requirements of the patients in each group. Given that the subjects were not controlled based on pathology or procedure type, there may be preferential use of tramadol over codeine in specific conditions or specific demographic groups, resulting in a mismatch between groups in terms of pain level. This is likely the cause of the age difference between groups. Nonetheless, the finding that tramadol prescriptions were refilled at a higher rate following surgery is significant.

Regardless of the presence of selection bias, the greater prescription MME of tramadol for older patients and the use of higher MME prescriptions for tramadol to nonoperative patients warrants further study. The findings of this study highlight the fact that codeine and tramadol are not prescribed as completely equivalent medications by hand surgeons. Further study is required to better understand prescriber attitudes toward the two medications, and whether these attitudes reflect the actual patient-reported pain control that they provide.

## Conclusions

In patients seen for upper extremity complaints by hand surgeons, total prescriptions MME for tramadol was than for codeine. In surgical patients, a higher rate of prescription refills was noted in patients given tramadol versus codeine despite initial equivalent MME prescription amounts. Nonsurgical patients prescribed tramadol were prescribed greater MME than those prescribed codeine. Patients who required prescription refills were prescribed larger initial doses in the tramadol group than those who were not prescribed a refill, but this finding was not seen in the codeine group.

Physician prescribing patterns differ between tramadol and codeine, with tramadol being prescribed in higher amounts to nonoperative and older patients. We find a need to better understand physician perceptions of the relative safety and efficacy of these two drugs in both the operative and nonoperative settings.
